# Individualized quality of life benefit and cost-effectiveness estimates of proton therapy for patients with oropharyngeal cancer

**DOI:** 10.1186/s13014-021-01745-1

**Published:** 2021-01-21

**Authors:** N. Patrik Brodin, Rafi Kabarriti, Clyde B. Schechter, Mark Pankuch, Vinai Gondi, Shalom Kalnicki, Madhur K. Garg, Wolfgang A. Tomé

**Affiliations:** 1 Institute for Onco-Physics, Albert Einstein College of Medicine, Bronx, NY 10461 USA; 2 Department of Radiation Oncology, Montefiore Medical Center, Bronx, NY 10461 USA; 3 Northwestern Medicine Chicago Proton Center, Warrenville, IL 60555 USA; 4 Department of Family and Social Medicine, Albert Einstein College of Medicine, Bronx, NY 10461 USA; 5 Department of Otorhinolaryngology - Head and Neck Surgery, Montefiore Medical Center, Bronx, NY 10461 USA; 6 Department of Urology, Montefiore Medical Center, Bronx, NY 10461 USA; 7 Department of Neurology, Albert Einstein College of Medicine, Bronx, NY 10461 USA

**Keywords:** Proton therapy, Quality of life, Cost-effectiveness, Individualized risk-assessment

## Abstract

**Background:**

Proton therapy is a promising advancement in radiation oncology especially in terms of reducing normal tissue toxicity, although it is currently expensive and of limited availability. Here we estimated the individual quality of life benefit and cost-effectiveness of proton therapy in patients with oropharyngeal cancer treated with definitive radiation therapy (RT), as a decision-making tool for treatment individualization.

**Methods and materials:**

Normal tissue complication probability models were used to estimate the risk of dysphagia, esophagitis, hypothyroidism, xerostomia and oral mucositis for 33 patients, comparing delivered photon intensity-modulated RT (IMRT) plans to intensity-modulated proton therapy (IMPT) plans. Quality-adjusted life years (QALYs) lost were calculated for each complication while accounting for patient-specific conditional survival probability and assigning quality-adjustment factors based on complication severity. Cost-effectiveness was modeled based on upfront costs of IMPT and IMRT, and the cost of acute and/or long-term management of treatment complications. Uncertainties in all model parameters and sensitivity analyses were included through Monte Carlo sampling.

**Results:**

The incremental cost-effectiveness ratios (ICERs) showed considerable variability in the cost of QALYs spared between patients, with median $361,405/QALY for all patients, varying from $54,477/QALY to $1,508,845/QALY between individual patients. Proton therapy was more likely to be cost-effective for patients with p16-positive tumors ($234,201/QALY), compared to p16-negative tumors ($516,297/QALY). For patients with p16-positive tumors treated with comprehensive nodal irradiation, proton therapy is estimated to be cost-effective in ≥ 50% of sampled cases for 8/9 patients at $500,000/QALY, compared to 6/24 patients who either have p16-negative tumors or receive unilateral neck irradiation.

**Conclusions:**

Proton therapy cost-effectiveness varies greatly among oropharyngeal cancer patients, and highlights the importance of individualized decision-making. Although the upfront cost, societal willingness to pay and healthcare administration can vary greatly among different countries, identifying patients for whom proton therapy will have the greatest benefit can optimize resource allocation and inform prospective clinical trial design.

## Background

Head and neck cancer (HNC) presents a major challenge in modern day radiation oncology, where radiation therapy (RT) is a critical component of an aggressive multidisciplinary treatment approach. Although effective as definitive therapy with 3-year loco-regional control rates in the range of 70–80% [1–6], HNC RT carries considerable risk for a large number of severe normal tissue complications. This substantial toxicity profile is usually ascribed to these cancers requiring very high doses of radiation and being anatomically situated in an area with many radiation-sensitive organs-at-risk (OARs). Thus, to deliver a sufficient radiation dose to the gross disease and involved lymph nodes the neighboring tissues will be subject to high doses of radiation. HNC constitutes a situation in which technological advances in RT have played a vital role in reducing the side effect profile, with the implementation of intensity-modulated RT (IMRT) being a key breakthrough.

The availability of proton radiation therapy constitutes another advance in the delivery of RT, and offers improved normal tissue sparing for a variety of cancer sites. The potential interest in HNC RT using protons is focused largely around reducing the risk of normal tissue toxicity. Proton therapy differs substantially from photon radiation therapy, characterized by much sharper distal dose fall-off, slightly wider penumbra, higher skin dose and different strategies to account for uncertainties in treatment delivery. Utilizing the proton therapy Bragg peak and a multi-beam approach, the goal is to reduce the dose to critical normal tissues, thereby reducing the risk of severe toxicity and improving the quality of life for HNC patients receiving definitive RT. Some of these strategies have been explored in previous modeling studies [7–11]. While results have been mixed as to the absolute benefit of proton therapy for HNC, an important remaining question is whether this benefit is also cost-effective, as proton therapy is currently expensive and of limited availability compared to standard photon RT.

We previously showed that the estimated quality of life benefit with proton therapy is highly variable between individual oropharyngeal HNC patients, suggesting that not all patients would receive a better treatment with protons [12]. Here, we performed a comprehensive cost-effectiveness analysis aiming to identify for which oropharyngeal cancer patients the benefit offered by proton therapy is estimated to be the most, or least, cost-effective.

## Methods and materials

### Patient material and quality-adjusted life years (QALYs) calculation

We previously published a quantitative decision-support strategy comparing the QALYs lost from normal tissue complications following definitive photon or proton RT for patients with oropharyngeal cancer [12]. Briefly, 33 oropharyngeal HNC patients treated with definitive photon IMRT at our institution were identified in accordance with an approved institutional review board protocol. Twenty-one patients were treated with bilateral comprehensive nodal irradiation and 12 patients with unilateral nodal irradiation. Comparative proton therapy treatment plans were generated based on clinical protocols from a collaborating institution using pencil beam scanning intensity-modulated proton therapy (IMPT) for HNC. Normal tissue complication probability (NTCP) models identified in a comprehensive systematic review [13] were used to calculate the estimated risk of dysphagia, esophagitis, xerostomia, hypothyroidism and oral mucositis for the photon and proton therapy plans, respectively.

Quality-adjustment factors (detailed in Additional file 1: Table S1) were then used to estimate the reduction in QALYs attributable to each complication, accounting for the age, sex and p16-status specific conditional survival probability. A detailed explanation of the proton therapy treatment planning along with NTCP and QALY calculations for these patients has been previously reported [12]. In short, the QALYs were calculated as the estimated survival time either free of any modeled normal tissue complications or spent in the corresponding health states of experiencing a given complication. Thus, the estimated QALYs depend on the probability of a given treatment complication, the estimated survival time, the duration of the given complication and the severity, which is represented by the quality-adjustment factors. A quality-adjustment factor (also referred to as utilities) of 1 would represent perfect health and 0 would represent death. This way, we can e.g. compare a mild complication that may last for a long time with a severe complication that only exists in an acute phase, using QALYs as a common scale metric.

### Cost-effectiveness analysis

The cost-effectiveness of proton IMPT compared to photon IMRT for oropharyngeal HNC RT was estimated based on 33 treatment fractions. The upfront cost of photon and proton therapy was determined from the 2017 CMS average national Medicare physician fee schedule reimbursement rates. Quoted costs of $18,415 for 30 fractions of complex IMRT and $27,772 for 25 fractions of complex proton therapy were extrapolated to $20,257 and $36,659 as the upfront cost of 33 fractions of IMRT and IMPT, respectively.

The interventions and patient procedures related to the management of the normal tissue complications included in this analysis are based on the standard of care for HNC patients treated at our institution. Patients with acute grade ≥ 3 oral mucositis receive long-acting pain medication in terms of a Fentanyl patch 25 μg/h and short-acting pain medication with 3–4 Percocet 325 mg tablets per day. They also receive a daily mucositis cocktail consisting of Lidocaine 2% viscous solution, Maalox dissolvable tablet and Benadryl 50 mg/ml and once weekly IV hydration for 4 weeks. Patients with acute grade ≥ 3 esophagitis receive similar management and a proportion of patients experiencing either of these side effects are also expected to require percutaneous endoscopic gastrostomy (PEG) tube placement (~ 30%), emergency room visits (~ 15% for oral mucositis and ~ 10% for esophagitis), and ~ 10% with these side effects require inpatient hospital admission. Patients with grade ≥ 3 oral mucositis or esophagitis are also expected to miss about 1 month of work due to these debilitating side effects.

Patients experiencing grade ≥ 2 dysphagia, which can be chronic in many cases, may require stricture dilation and this proportion was estimated to be about 15–17% of HNC patients according to previous studies [14, 15]. A proportion of patients with grade ≥ 2 dysphagia also require a chronic PEG tube (~ 10%). Patients experiencing chronic hypothyroidism (defined as elevated TSH w/ or w/o T4/T3 changes requiring hormone replacement) will receive lifelong Levothyroxine at 1.6 μg/kg daily as recommended by Vaidya and Pearce [16]. We currently do not have an effective standard of care management for grade ≥ 2 xerostomia and this is considered a chronic condition. For this analysis we used the suggested intervention from Siddiqui and Movsas of Pilocarpine 5 mg three times per day for 8–12 weeks and Cevimeline 30–45 mg three times per day for 6 weeks [17].

The costs of any drug treatments were extracted from the US National Average Drug Acquisition Cost (NADAC) database (accessed March 2018) and costs for medical procedures, emergency room visits and inpatient hospitalization were extracted from US national statistics or published reports, summarized in Table 1. The cost of lost income was based on the US median income per capita from 2018 census bureau statistics. Discounting was applied for the estimated QALYs, as well as cost of Levothyroxine therapy and chronic PEG tube dependency, as these require long-term management. A standard discounting rate of 3% per year was used. All costs were standardized to 2018 estimates as a common timeframe.

**Table 1:** The management and patient procedures related to the different normal tissue complications, as well as the estimated total cost of the different parts of managing the complications or the cost of the related patient procedure

Normal tissue complication	Management/patient procedure	Estimated cost	Reference
Oral mucositis (grade ≥ 3) or Esophagitis (grade ≥ 3)	Fentanyl 25 μg/h patch (for 6 weeks)	$168.8	NADAC database
	Percocet 325 mg tablet (for 6 weeks)	$1514.1	NADAC database
	Mucositis cocktail (for 6 weeks)	$37.0	NADAC database
	Weekly IV hydration (for 4 weeks)	$154.2	2019 Medicare Coding and Payment Report
	PEG tube placement in 30% of cases	$5686*	Callahan et al. [18]
	Emergency room visit in 15% of cases for oral mucositis and 10% of cases for esophagitis	$2096	2018 Health Care Cost and Utilization Report^†^
	In patient hospitalization in 10% of cases	$19,672	2018 Health Care Cost and Utilization Report
	Loss of 1 month of work	$2718	US Census Bureau Median per Capita Income 2014–2018^‡^
Dysphagia (grade ≥ 2)	Chronic PEG tube in 10% of cases	$18,836/year*	Callahan et al. [18]
	Stricture dilation in 16% of patients [14, 15]	$1700 (based on average Medicare charges ranging from $1200 to $2200)	www.howmuchisit.org/esophageal-dilation-cost/ (updated Aug 2018)
Hypothyroidism (elevated TSH with or without T4/T3 changes requiring hormone replacement)	Levothyroxine hormone replacement	$174.2/year (assuming 70 kg body weight)	NADAC database
Xerostomia (grade ≥ 2)	Pilocarpine 5 mg (for 10 weeks)	$231	NADAC database
	Cevimeline 30-45 mg (for 6 weeks)	$251.4	

^*^Adjusted for inflation from 1998 costs presented in the referenced paper to 2018 dollars

^†^https://healthcostinstitute.org/images/pdfs/HCCI_2018_Health_Care_Cost_and_Utilization_Report.pdf

^‡^https://www.census.gov/quickfacts/fact/table/bronxcountybronxboroughnewyork,US/INC110218

The total costs including upfront RT and the cost of normal tissue complication management were calculated for 10,000 Monte Carlo samples for each of the 33 patient cases according to:

1 $$\begin{aligned} Cos{t_{Total,Photon}} & = Cos{t_{RT,Photon}} + \sum\limits_i {Cos{t_{Management,i}}Even{t_{i,Photon}}} {P_{Management,i}} \\ Cos{t_{Total,Proton}} & = Cos{t_{RT,Proton}} + \sum\limits_i {Cos{t_{Management,i}}Even{t_{i,Proton}}} {P_{Management,i}} \\ \end{aligned}$$

where *i* represents each of the modeled normal tissue complications, *Event* represents whether or not that complication occurred in each Monte Carlo sample and *P*_*Management*_ represents the proportion a certain management would be employed for complication *i*. For example, Levothyroxine would be employed in 100% of hypothyroidism cases whereas PEG tube placement is modeled to be needed in 30% of grade ≥ 3 oral mucositis or esophagitis cases. For hypothyroidism and chronic PEG tube dependency following dysphagia, the cost of management is integrated over the patient’s remaining life expectancy. Once the total costs and estimated QALYs for proton and photon therapy were calculated, incremental cost-effectiveness ratios (ICERs) for each sampled case were generated as:

2 $$ICER = \frac{{Cos{t_{Total,Proton}} - Cos{t_{Total,Photon}}}}{{QAL{Y_{Proton}} - QAL{Y_{Photon}}}}$$

As such, ICERs represent the cost of sparing one full QALY with proton therapy. For cases with a zero or negative QALY difference photon therapy “dominates”, and conversely, if the QALY difference is positive and the total cost of proton therapy is less than that of photon therapy, proton therapy dominates.

### Sensitivity analyses

A one-way sensitivity analysis was performed to assess the impact of the assumed proportion of patients for whom dysphagia was chronic, or cleared up within 5 years in the QALY calculation. In the base case dysphagia was assumed chronic in 50% of cases and resolved within 5 years in 50%. This was varied between respectively 0% and 100% in the sensitivity analysis.

We also performed a one-way sensitivity analysis of the cost of proton therapy to determine the impact on the cost-effectiveness results. In addition, a full sensitivity analysis was performed by varying the critical assumptions of the different components throughout the QALY and cost-effectiveness calculation steps as detailed in Table 2.

**Table 2:** One-way and full sensitivity analyses performed as part of the cost-effectiveness evaluation

Component	Base case	Variation in sensitivity analysis
**One-way sensitivity analysis**		
Proportion of chronic dysphagia	50% chronic, 50% resolved within 5 years	Scenario 1: 0% chronic, 100% resolved within 5y Scenario 2: 100% chronic, 0% resolved within 5y
Cost of proton therapy (33 fx)	$36,659	Scenario 1: $31,659 Scenario 2: $26,659 Scenario 3: $21,659
**Full sensitivity analysis**		
* QALY calculation*		
Hazard ratio for patients with > 10 pack-year smoking history	1.73	Normal distribution matching 95% CI 1.17–2.57
Quality-adjustment factors		Beta distributions matching 95% CIs:
Dysphagia (grade ≥ 2)	0.83	0.70–0.93
Esophagitis (grade ≥ 3)	0.66	0.35–0.90
Xerostomia (grade ≥ 2)	0.82	0.72–0.90
Hypothyroidism (elevated TSH with or without T4/T3 changes requiring hormone replacement)	0.97	0.94–0.98
Oral mucositis (grade ≥ 3)	0.06	0.01–0.15
* Cost-effectiveness calculation*		
Proportion of oral mucositis or esophagitis cases requiring inpatient hospital admission	10%	Normal distribution matching 95% CI 0–20%
Proportion of oral mucositis cases resulting in emergency room visit	15%	Normal distribution matching 95% CI 5–25%
Proportion of esophagitis cases resulting in emergency room visit	10%	Normal distribution matching 95% CI 0–20%
Proportion of dysphagia cases requiring chronic PEG tube	10%	Normal distribution matching 95% CI 0–20%
Proportion of patients receiving stricture dilation for dysphagia	16%	Normal distribution matching 95% CI 11–21%
Cost of stricture dilation	$1700	Normal distribution matching 95% CI $1200–$2200

More information about the components used in the quality-adjusted life years calculation can be found in ref [12]

## Results

### Base case analysis

Incremental cost-effectiveness ratios (ICERs) were calculated for each of the 10,000 Monte Carlo samples for 33 individual patients, resulting in 330,000 total samples. In the base case, there is considerable variation in the calculated ICERs between patients, with median $361,405/QALY (IQR: $45,453/QALY–$1,556,948/QALY) for the entire cohort. The median ICER for patients < 65 years old is $341,081/QALY, compared to $399,533/QALY for those who were ≥ 65. There is a considerable difference in ICER based on p16-status with median $516,297/QALY for patients with p16-negative tumors and $234,201/QALY for those with p16-positive tumors.

**Fig. 1:**
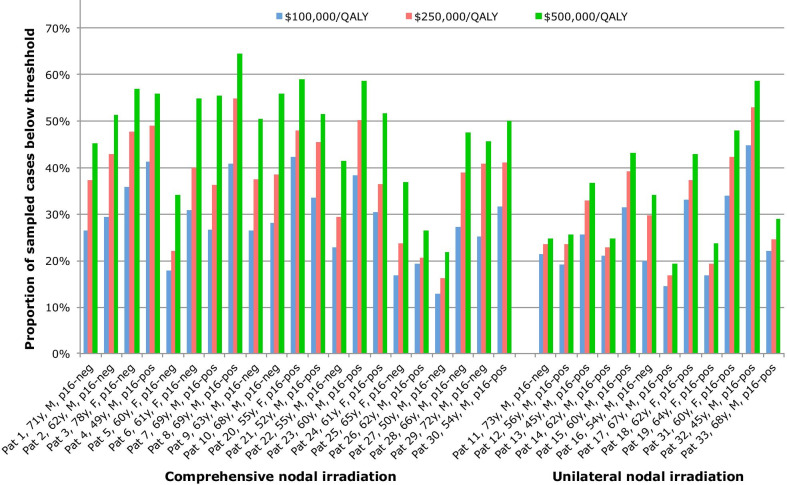
The proportion of sampled cases that would be considered cost-effective based on three different thresholds are shown for each individual patient, with patients receiving comprehensive nodal irradiation on the left-hand side, and unilateral nodal irradiation on the right-hand side

The variation in cost-effectiveness of proton therapy between patients is shown in Fig. 1, where the proportion of ICERs falling below certain cost-effectiveness thresholds are displayed, along with given patient characteristics. A larger proportion of sampled cases are considered cost-effective for patients receiving comprehensive nodal irradiation, compared to unilateral neck irradiation. This is related to 41.7% of sampled cases showing no difference in the estimated QALYs between photon and proton therapy for patients receiving unilateral nodal irradiation, as the risk of normal tissue complications is lower in this group. This is attributed to the lower risk of experiencing any of the modeled treatment complications (because of less normal tissue irradiated during unilateral treatment), leading to a lower likelihood that proton therapy would be cost-effective for these patients.

Our results show that proton therapy is more likely to be considered cost-effective for patients with p16-positive tumors treated with comprehensive nodal irradiation, with 42.4% of sampled cases considered cost-effective at a threshold of $250,000/QALY, compared to 26.6% of cases for those with p16-negative tumors treated with unilateral nodal irradiation. The variation between patients is further highlighted as the median ICER for the patient where proton therapy is most likely to be cost-effective is $54,477/QALY, compared to $1,508,845/QALY for the patient where proton therapy is least likely to be cost-effective.

### Sensitivity analyses

Varying the assumption of dysphagia being chronic or cleared within 5 years did not substantially impact the results, with 35.2% of sampled cases considered cost-effective at $250,000/QALY in the base case, compared to 34.8% if resolved within 5 years and 35.6% if chronic.

Conversely, varying the cost of proton therapy had a significant impact on the results with a median ICER of $361,405/QALY in the base case, compared to $244,351/QALY, $133,234/QALY and $26,394/QALY for an upfront proton therapy cost of $31,659, $26,659 and $21,659, respectively. Similarly, the variation in proportion of sampled cases that would be cost-effective at different thresholds varies with proton therapy cost, as shown in Table 3.

**Table 3:** The proportion of sampled cases that are considered cost-effective at different thresholds and varying cost of proton therapy for the whole patient cohort (for the patient where proton therapy is least likely to be cost-effective on the left-hand side of the parentheses, and most likely to be cost-effective on the right-hand side)

Cost-effectiveness threshold	Proton therapy cost			
	Base case: $36,659	$31,659	$26,659	$21,659
$100,000/QALY	27.5% (21.3, 44.9%)	32.6% (22.7, 51.3%)	36.4% (24.0, 54.7%)	56.0% (34.6, 66.8%)
$250,000/QALY	35.2% (23.4, 53.0%)	39.4% (24.2, 56.2%)	47.0% (26.3, 61.4%)	65.7% (51.8, 70.8%)
$500,000/QALY	43.2% (24.7, 58.6%)	48.4% (27.2, 62.3%)	56.5% (34.7, 67.1%)	70.5% (61.8, 72.5%)

In the base case, for patients with p16-positive tumors treated with comprehensive nodal irradiation proton therapy is estimated to be cost-effective in ≥ 50% of sampled cases for 8/9 patients at $500,000/QALY, compared to only 6/24 patients who either have p16-negative tumors or receive unilateral irradiation. If the upfront cost of proton therapy was $31,659 then this would instead be 8/9 compared to 11/24, respectively.

**Fig. 2:**
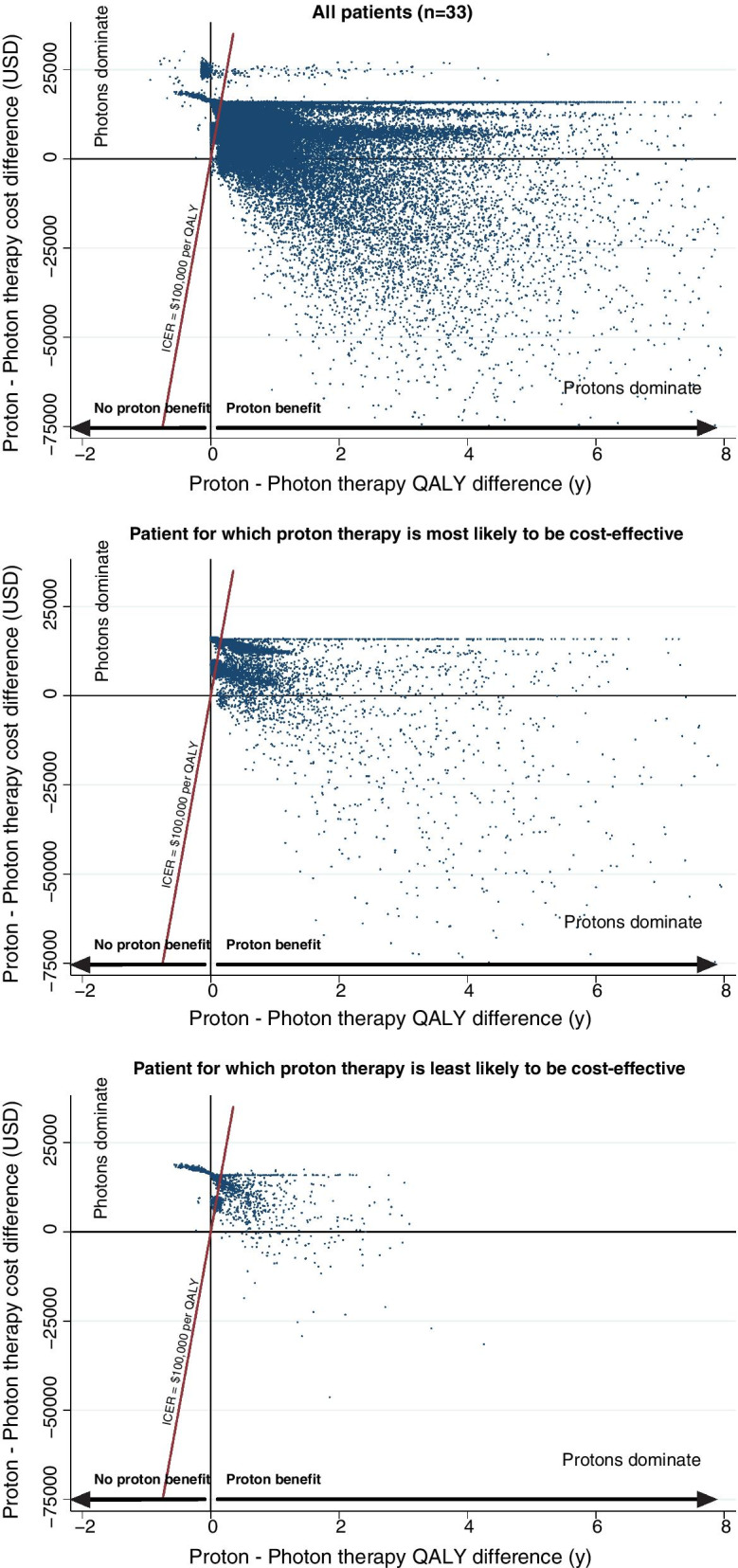
The full sensitivity analysis showing cost and QALY differences between proton and photon therapy for all patients as well as for the patients for which proton therapy is most and lest likely to be cost-effective, respectively. Simulated cases to the right of the red line would be considered cost-effective at $100,000/QALY whereas cases to the left would not

The results of the full sensitivity analysis are shown in Fig. 2 as the variation in total cost difference and QALY difference between proton and photon therapy for each of the Monte Carlo sampled cases. This shows a large variation in the sampled cases with a considerable proportion of data points to the right of the line that indicates an ICER equal to $100,000/QALY, indicating proton therapy as cost-effective. The majority of data points are bunched together at a level closer to the $100,000/QALY line, where the estimated benefit from proton therapy is between 0 to 1.0 QALYs. In 22.9% of cases photon therapy dominates, i.e. there is no estimated benefit from proton therapy, mainly derived from sampled cases with no expected normal tissue complications. On the other hand, proton therapy dominates in 2.7% of sampled cases, where proton therapy has both a QALY benefit and are estimated to have a lower total cost compared to photon therapy. The two example patient cases in Fig. 2 where proton therapy is most likely or least likely to be cost-effective illustrate that for certain oropharyngeal cancer patients there is a considerable benefit from proton therapy with a majority of sampled cases to the right of the $100,000/QALY line, and conversely, little expected benefit for other patients.

## Discussion

Similar to the expected quality of life benefit, here we have shown that the cost-effectiveness of proton therapy varies strongly between individual patients. Proton therapy is estimated to be more cost-effective for patients with p16-positive tumors, and those treated with comprehensive nodal irradiation. This suggests that proton therapy would be most beneficial for oropharyngeal cancer patients who are expected to be at high risk of experiencing treatment-related normal tissue complications, and those who have a more promising cancer prognosis and therefore longer life expectancy. This type of model-based approach provides an avenue for patient selection based on estimated clinical benefit, which can be weighted against the increased cost of treatment. This approach could also allow for testing the estimated benefits as patients are selected for one treatment vs. another, and could be re-calibrated as more clinical data becomes available.

The applied methodology estimates the cost-effectiveness of proton therapy based on dosimetric differences and corresponding NTCP estimates which then inform calculations on QALYs. Even when including the uncertainty in NTCP model parameters through all parts of the QALY calculation chain, the main factor driving the cost-effectiveness is the upfront cost of proton therapy. Clearly, lowering the cost to a level closer to that of current photon IMRT would generate more scenarios in which proton therapy is cost-effective at lower thresholds. Importantly though, for sub-groups of patients such as those with p16-negative tumors treated with unilateral neck irradiation, proton therapy may not be cost-effective even at a lower cost, as the estimated quality of life benefit is minimal for some of these patients.

In comparison to our results, Cheng et al. found that proton therapy delivered with IMPT would be cost-effective at €80,000 per spared QALY for 8 of 23 HNC patients [7]. The reduction in the estimated risk of xerostomia and dysphagia they found with proton therapy is similar to our study, but the difference in upfront cost of the two treatments was somewhat smaller in their analysis. Conversely, a study by Ramaekers et al. [10] found that proton therapy was not cost-effective for HNC patients compared to photon IMRT. In their analysis they found an estimated average 0.10 QALYs spared with proton therapy, which is less than the average 0.37 QALY difference previously reported for our patient cohort [12]. This may be related to differences in the study populations or the fact that they only estimated xerostomia and dysphagia at 12 months as their endpoints, whereas we considered the risk of xerostomia, dysphagia, esophagitis, hypothyroidism and oral mucositis in this analysis.

One should also consider that both the upfront treatment cost and the costs related to managing complications may vary considerably between different countries and institutions. Thus the focus of this study was not to determine whether or not proton therapy is cost-effective in general but to highlight the variability in estimated benefit and cost-effectiveness between individual patients, warranting individualized assessments when deciding whether to offer proton therapy. Another important limitation of this study is that not all possible treatment complications can be quantitatively modeled. Therefore, the estimates provided here may somewhat underestimate the cost-effectiveness of proton therapy, if the risks of non-modeled side effects are also reduced due to the lower integral dose from proton therapy. Based on the high costs of unplanned emergency room visits and hospitalizations, reducing these may be one of the key components of proton therapy cost-effectiveness. This is supported by a recent propensity-matched analysis showing reduced rates of severe toxicity and lower rates of unplanned hospitalizations in patients treated with proton therapy, compared to contemporary photon therapy [19].

Common to our study as well as those by others is the assumption that proton and photon therapy has the same treatment efficacy and probability of disease control. This is a critical assumption, as a reduction in loco-regional control would outweigh any benefit the patient receives from an improvement in OAR sparing. Encouragingly, results from preliminary clinical studies support this assumption of equal treatment efficacy between photon IMRT and proton IMPT [20, 21]. Key to this assumption is timely delivery of proton therapy, as delays in the time from diagnosis to treatment would negatively impact treatment outcome [22]. For the estimated benefit of proton therapy to withstand, efforts should be made to overcome existing logistical challenges related to proton therapy, in order to avoid delays in treatment delivery as compared to standard photon IMRT. Another recent study compared NTCP estimates between IMRT and pencil beam scanning proton therapy for a cohort of 30 oropharyngeal cancer patients [23]. Similar to our results they found that certain patients are expected to benefit greatly from proton therapy, with ~ 15% reduction of xerostomia or dysphagia, whereas for others there was close to no difference in NTCP estimates between photons and protons.

## Conclusions

Our results suggest that individualized decision-making is key when deciding whether to offer proton therapy for oropharyngeal cancer, which is supported by results from other studies as well. This strengthens the conclusion that proton therapy is unlikely to be cost-effective for all oropharyngeal cancer patients receiving definitive RT, and highlights the need to identify the patients with the most to gain from this treatment option. Furthermore, there is a difference in costs, societal willingness to pay and health system administration among different countries and this also needs to be considered when making decisions about which patients should receive proton therapy. Model-based decision-support systems and cost-effectiveness analyses such as the one presented here can help identify the patients for whom proton therapy is most likely to be beneficial, and serve as a decision-making aid for clinicians and health systems.

## Supplementary Information

**Additional file 1**. Supplementary tables and figures.

## Data Availability

The datasets used and/or analyzed during the current study are available from the corresponding author on reasonable request.

## Abbreviations

HNC: Head and neck cancer
RT: Radiation therapy
OARs: Organs at risk
IMRT: Intensity-modulated radiation therapy
QALYs: Quality-adjusted life years
IMPT: Intensity-modulated proton therapy
NTCP: Normal tissue complication probability
PEG: Percutaneous endoscopic gastrostomy
TSH: Thyroid stimulating hormone
NADAC: National Average Drug Acquisition Cost
ICERs: Incremental cost-effectiveness ratios
