# Supplementary material for: Individualized quality of life benefit and cost-effectiveness estimates of proton therapy for patients with oropharyngeal cancer
Source: Radiat Oncol. 2021 Jan 21;16:19. doi: 10.1186/s13014-021-01745-1 (PMC7819210; doi:10.1186/s13014-021-01745-1)
Supplement: Supplementary file 1 — Additional file 1. Supplementary tables and figures. [file 13014_2021_1745_MOESM1_ESM.docx]

**Table S1.** Quality-adjustment factors utilized for modeled normal tissue complications. Simulated parameter distributions were used as input for the quality-adjusted life years (QALY) analysis in order to account for statistical uncertainty in the quality-adjustment factors.

| **Endpoint** | **Quality-adjustment factor** | **Reference** |
| --- | --- | --- |
| ***Dysphagia (grade ≥2)*** | 0.85 ± 0.11 | Rudmik et al. 2015 (PMID: 26171771) |
|  | 0.803 ± 0.14 | Ramaekers et al. 2011 (PMID: 21683647) |
|  | **Parameter distribution**  **Mean 0.83  (95% CI: 0.70 – 0.93)** |  |
| ***Esophagitis***  ***(grade ≥3)*** | 0.82  (range: 0.5 – 0.95) | Heudebert et al. 1997 (PMID: 9097989) |
|  | 0.493 ± 0.075 | Ramaekers et al. 2013 (PMID: 24457241) |
|  | **Parameter distribution**  **Mean: 0.66  (95% CI: 0.35 – 0.90)** |  |
| ***Hypothyroidism***  ***(TSH w/ or w/o T4/T3 changes requiring hormone replacement)*** | 0.99  (range: 0.9 – 0.99) | Garcia et al. 2014 (PMID: 24862564) |
|  | 0.99  (range: 0.9 – 0.99) | Lee et al. 2014  (PMID: 25444226) |
|  | 0.99  (range: N/A) | Vidal-Trecan et al. 2002  (PMID: 11888833) |
|  | 0.90  (range: N/A) | Lundkvist et al. 2005 (PMID: 16332592) |
|  | **Parameter distribution**  **Mean: 0.97  (95% CI: 0.94 – 0.98)** |  |
| ***Xerostomia***  ***(grade ≥2)*** | 0.817 ± 0.19 | Ramaekers et al. 2011 (PMID: 21683647) |
|  | 0.827  (range: 0.819 – 0.841) | Kohler et al. 2013  (PMID: 24138916) |
|  | **Parameter distribution**  **Mean: 0.82  (95% CI: 0.72 – 0.90)** |  |
| ***Oral mucositis  (grade ≥3)*** | 0.061  (range: N/A) | Chan et al. 2011  (PMID: 21744880) |
|  | 0.062  (range: N/A) | Brown et al. 2008 (PMID: 18194407) |
|  | **Parameter distribution**  **Mean: 0.06  (95% CI: 0.01 – 0.15)** |  |

**Table S2.** Patient characteristics.

| **Patient characteristics** | **All patients**  **(*n* = 33)** |
| --- | --- |
| Age (y), mean±SD | 61 ± 8 |
| Sex, n (%)  Male  Female | 24 (73%)  9 (27%) |
| Stage, n (%)  II  III  IVa | 8 (24%)  5 (15%)  20 (61%) |
| HPV p16-status, n (%)  Negative  Positive | 14 (42%)  19 (58%) |
| Highest RT dose level, n (%)  <66 Gy  66 Gy  >66 Gy | 7 (21%)  5 (15%)  21 (64%) |
| Chemotherapy, n (%)  Concurrent  None | 25 (76%)  8 (24%) |
| Smoking status, n (%)  >10 pack-years  ≤10 pack-years | 23 (70%)  10 (30%) |
